# CIDACS-RL: a novel indexing search and scoring-based record linkage system for huge datasets with high accuracy and scalability

**DOI:** 10.1186/s12911-020-01285-w

**Published:** 2020-11-09

**Authors:** George C. G. Barbosa, M. Sanni Ali, Bruno Araujo, Sandra Reis, Samila Sena, Maria Y. T. Ichihara, Julia Pescarini, Rosemeire L. Fiaccone, Leila D. Amorim, Robespierre Pita, Marcos E. Barreto, Liam Smeeth, Mauricio L. Barreto

**Affiliations:** 1 Centre for Data and Knowledge Integration for Health (CIDACS), Fiocruz Bahia, Parque Tecnológico da Bahia, Edf. Tecnocentro, sala 315, Rua Mundo, no 121, Salvador, 41301-110 Brazil; 2 Department of Non-communicable Disease Epidemiology, London School of Hygiene and Tropical Medicine, London, UK; 3 NDORMS, Center for Statistics in Medicine, University of Oxford, Oxford, UK; 4 Department of Statistics, Federal University of Bahia (UFBA), Salvador, Brazil; 5 Institute of Public Health, Federal University of Bahia (UFBA), Salvador, Brazil; 6 Computer Science Department, Federal University of Bahia (UFBA), Salvador, Brazil; 7 Department of Statistics, London School of Economics and Political Science (LSE), London, UK

**Keywords:** Accuracy, Data linkage, Entity resolution, Indexing, Information retrieval techniques, Scalability, Scoring Search

## Abstract

**Background:**

Record linkage is the process of identifying and combining records about the same individual from two or more different datasets. While there are many open source and commercial data linkage tools, the volume and complexity of currently available datasets for linkage pose a huge challenge; hence, designing an efficient linkage tool with reasonable accuracy and scalability is required.

**Methods:**

We developed CIDACS-RL (Centre for Data and Knowledge Integration for Health – Record Linkage), a novel iterative deterministic record linkage algorithm based on a combination of indexing search and scoring algorithms (provided by Apache Lucene). We described how the algorithm works and compared its performance with four open source linkage tools (AtyImo, Febrl, FRIL and RecLink) in terms of sensitivity and positive predictive value using gold standard dataset. We also evaluated its accuracy and scalability using a case-study and its scalability and execution time using a simulated cohort in serial (single core) and multi-core (eight core) computation settings.

**Results:**

Overall, CIDACS-RL algorithm had a superior performance: positive predictive value (99.93% versus AtyImo 99.30%, RecLink 99.5%, Febrl 98.86%, and FRIL 96.17%) and sensitivity (99.87% versus AtyImo 98.91%, RecLink 73.75%, Febrl 90.58%, and FRIL 74.66%). In the case study, using a ROC curve to choose the most appropriate cut-off value (0.896), the obtained metrics were: sensitivity = 92.5% (95% CI 92.07–92.99), specificity = 93.5% (95% CI 93.08–93.8) and area under the curve (AUC) = 97% (95% CI 96.97–97.35). The multi-core computation was about four times faster (150 seconds) than the serial setting (550 seconds) when using a dataset of 20 million records.

**Conclusion:**

CIDACS-RL algorithm is an innovative linkage tool for huge datasets, with higher accuracy, improved scalability, and substantially shorter execution time compared to other existing linkage tools. In addition, CIDACS-RL can be deployed on standard computers without the need for high-speed processors and distributed infrastructures.

## Background

Linking records from big health and non-health related administrative data sources has been popular in many countries, including Australia, Brazil, Canada, United Kingdom, and the USA. It overcomes the limitations of using an isolated data source and has contributed significantly to the advancement of knowledge [1–4], health service research [5, 6], changes in clinical practice [1–3], health policy reforms [3, 5, 7, 8], and elaboration of social actions and policies to reduce poverty and social inequalities [9–12].

Record linkage, also called data linkage, is the process of combining records about the same individual or entity from two or more different data sources [13, 14]. It can also be defined as the process of identifying duplicate records in the same dataset [14]. In principle, a record linkage problem comprises the need of comparing pairs from different datasets and classifying such pairs as “linked” or “non-linked” with reasonable accuracy [15]. It enables integrate data from multiple data sources, thereby supplementing information on an individual (for example), the validation of information collected in one data source [15] or the de-duplication of records within a single data source [13, 14]. Record linkage also has additional applications such as building longitudinal profiles of individuals and case-identification in capture-recapture studies [16].

There are two main types of linkage algorithms: deterministic and probabilistic. Deterministic linkage methods vary from a one-step procedure using a single unique identifier or a set of several attributes (called “exact deterministic linkage”) to step-wise algorithmic linkages involving a series of progressively less restrictive steps to allow variations between record attributes (called “iterative deterministic linkage”). A record pair is classified as “linked” if it meets the criteria or parameters at any step; otherwise it is classified as “non-linked” [17]. Probabilistic linkage methods, on the other hand, take advantage of differences in the discriminatory power of each attribute, and apply calculation of similarity scores and decision rules using thresholds to classify record pairs as “linked”, “potentially linked” (treated as “dubious” records in most linkage tools) and “non-linked” [17, 18]. It was introduced by Newcombe [19] and mathematically formalised by Fellegi and Sunter [20]. It accounts for some inconsistencies in the linking fields such as typographical errors in names using partial agreement weights and some levels of missing data by applying standard multiple imputation techniques [17, 18, 21].

Several variations of record linkage methods and computerized tools in the literature, have emerged to meet different requirements and challenges, such as accuracy, speed, and scalability. Many of these tools have a general purpose, allowing a combination of existing configurations and methodologies [22–27]. While most of these methods are probabilistic, some of them apply a combination of deterministic and probabilistic linkages (hence called “hybrid” methods) [27]. In general, a successful linkage procedure involves five main steps: (i) pre-processing, (ii) some form of blocking, (iii) pair-wise comparison, (iv) classification of the pairs in “linked” and “non-linked”, and v) accuracy assessment [25].

The pre-processing step involves data cleaning and standardization whereby incomplete and incorrectly formatted data is converted into a well-defined, consistent form [24, 25]. Specific approaches to deal with missing data can be applied to i) remove missing fields or entire records or ii) impute missing values based on standard or calculated values. Pre-processing may also involve anonymization techniques, such as Bloom filters [15, 27], to protect sensitive data from disclosure and unauthorized use.

Executing a linkage routine between data sets A and B will result in $|A| * |B|$ comparisons. In a big data context, these numbers make peer-to-peer comparisons impractical and lead to a number of infrastructure, data processing, and data analysis challenges [28, 29]. To circumvent scalability challenges over big datasets, different approaches have been used, including parallelism or distribution using multi-core processors, cluster-based platforms, or graphics processing units (GPUs) [27, 30], and blocking (and/or indexing) strategies, as well as their combinations [25, 27, 31].

The blocking and indexing step generates pairs of candidate records pertaining to the same comparison block [31]. These methods drastically decrease the number of candidate record pairs to a feasible number thereby speeding up the linkage performance over big datasets while still maintaining linkage accuracy. Several indexing techniques that have been used in record linkage are described in the literature [31].

The field comparison and classification step measures the similarity of attributes for each record pair using different functions, hence calculates a matching weight. This step classifies candidate record pairs using weight vectors as “matches”, “non-matches”, and “possible matches”. The “posible matches” group can be manually assessed and further classified into “matches” or “non-matches” using a clerical or manual review process [25, 31]. The choice of similarity functions depends on the content of the field: string comparison functions are used for names and addresses whereas numerical comparison functions are used for fields such as date, age and numerical values [31]. The weight vector for each record pair is formed by combining all the matching weights calculated by the different comparison functions [25, 31] and is compared against one or more cut-off (threshold) points, depending on the decision model used.

The final step–accuracy assessment–evaluates the linkage algorithm and the quality of the linkage (i.e. it estimates rates of linkage errors: missed matches and false matches). Linkage accuracy is often assessed using a gold standard dataset where the true match status of each pair of records is known [24, 25]. Comparing the probabilistically linked dataset to the gold-standard dataset will identify true matches, true non-matches, false matches, and missed matches. Hence, measures of linkage quality such as sensitivity, positive predictive value, and F-measure can be easily derived. When a gold standard dataset is not available, alternative approaches such as sensitivity analysis, comparison of characteristics of linked and non-linked data, and identification of implausible matches could be used to quantify the rate of linkage errors [29].

In this paper, we aim to describe the architecture and design CIDACS-RL, a record linkage tool that utilizes a novel application of combined indexing search and scoring algorithms (provided by Apache Lucene). In addition, we provide evidence on its accuracy and scal-ability compared to existing open source linkage tools. The motivation behind the development of CIDACS-RL arise from the need to link huge datasets in the range of millions in a reasonable time for which the other tools in this comparison are not designed. Both indexing and scoring algorithms have long been used in many different application domains including record linkage. However, to our knowledge, the combined implementation of indexing search and scoring (such as inverted index and term frequency inverse document frequency, TF-IDF) as a blocking procedure, using Apache Lucene [32], with high accuracy and scalability in linking huge datasets is novel.

This paper is structured as follows: “Methods” section presents the architecture and design of CIDACS-RL, as well a summary of other data linkage tools. This section also describes our methodological approach and the datasets used in this study. Accuracy and scalability results obtained with a real case study and a simulated cohort are presented in “Results” section and a further discussion is provided in “Discussion” section. Finally, “Conclusion” section concludes the paper highlighting our contributions and potential future development on the linkage algorithms.

## Methods

### CIDACS record linkage tool

CIDACS-RL is a tool developed at the Centre for Data and Knowledge Integration for Health (CIDACS) to link administrative electronic health records and socioeconomic datasets hosted at the centre. All datasets are identifiable at individual level and cover a significant period of time (from 2003 to 2018), storing data on demographics, health episodes, and participation in social protection programmes in thousands of records. The main dataset (CadUnico) has more than 100 million records and is the main baseline for linking to other health and socioeconomic datasets to produce bespoke data (“data marts”) for epidemiological analysis. Detailed description of data sources in Brazil used in linkage for epidemiological studies can be found elsewhere [33].

Linkage imposes a significant challenge in terms of execution time: from one day to one week, depending on the databases. Besides feasible execution times, CIDACS-RL also aims to achieve high accuracy (high positive predictive value). This is because the linked dataset is mostly used for epidemiological studies to evaluate associations between exposures and health outcomes, and potential sources of bias due to linkage should be avoided [29]. Within the CIDACS environment, all datasets are submitted to data cleaning and quality assurance processes before entering the data linkage steps. These processes ensure that linkage attributes are standardized and cleaned, to the best of our ability.

To address the execution requirement, CIDACS-RL makes use of combined indexing search and scoring algorithms provided by Apache Lucene [32], an open source software with a full-featured text search engine library. These algorithms are used as a blocking step to reduce the number of comparisons during the linkage. If two databases *A* and *B* were to be linked and $|\cdot |$ denotes the number of records in a given database, assuming $|A| > |B|$ (i.e., *A* is the largest database), CIDACS-RL indexes the largest database (*A*) and then each record in *B* is searched based on this index. Thus, instead of comparing each record of *B* with all records of *A*, only a small portion of *A* is compared.

**Fig. 1:**
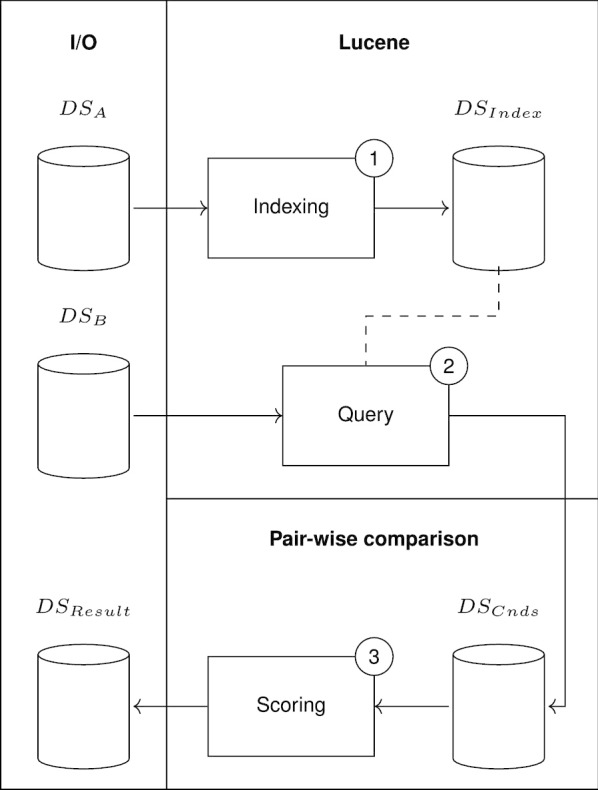
CIDACS-RL Architecture. In the first step the dataset A is indexed. During the query step, each record in dataset B is used to retrieve the *N* most similar records in the index and store them in a logical dataset (candidates). Finally, in the query step, a custom comparison method is applied to classify the candidate pairs

CIDACS-RL architecture is presented in Fig. 1. Indexing and Query modules (based on Lucene library) are used in the blocking step. The blocking process takes into account only the dataset *A* ($DS_A$), which is read by the I/O module. The pairwise comparison layer reads candidate pairs, i.e., dataset Cnds ($DS_{Cnds}$), and each record from dataset B ($DS_B$) is used to query similar records from indexed dataset *A* ($DS_{Index}$). The scoring module is then used to compare the candidate pairs, and the result ($DS_{Result}$) is written by the I/O module.

#### Indexing and query processes

The Indexing module take as input the linkage attributes from dataset *A* (the larger dataset) and builds an index *Ai*. CIDACS-RL uses Lucene’s tools during the indexing processes: strings are stored as text fields and written to the index. Through the I/O module, CIDACS-RL reads each line in dataset *A*, creates one instance of text field class for each attribute, and writes the attribute set corresponding to each line to a Lucene document.

A challenging issue in linking huge datasets is to reduce the number of pairwise comparisons. Blocking strategies used for this purpose must be carefully developed, as they have a direct impact on the final result. This is because blocking restricts the comparisons made by the linkage system - true records might not be compared if the blocking process is too restrictive or if there are any errors on the variables used for blocking [29]. Therefore, the query module is used in CIDACS-RL in the blocking step. Instead of comparing each record from dataset *B* with every record from dataset *A*, we query a small subset of similar records from *Ai* and apply comparison functions on them. CIDACS-RL uses a mixture of exact, semi-exact, and fuzzy queries provided by Apache Lucene to overcome different errors that may exist in data linkage attributes, such as missing or duplicate characters and abbreviations.

The Query module performs the query in three different ways: (i) exact, (ii) semi-exact, and (iii) fuzzy. Exact query takes each linkage attribute as a parameter and returns only records in which every attribute is equal to those used for querying. Semi-exact query is a modification of exact query being composed of an arrangement of $n-1$ linkage attributes. This arrangement aims to retrieve candidate pairs where only one attribute is different between the query record and the resulting pair. Unlike exact and semi-exact queries, fuzzy query allows differences on any number of attributes. Fuzzy queries have a higher average running time than exact queries: 67.5 milliseconds versus 2.1 milliseconds, respectively, over 114 million records. To reduce the overall running time when using a mixture of exact and fuzzy queries, all exact and semi-exact queries are performed first since they demand lesser computational time.In our comparison, fuzzy simply means that we matched records that are not exactly equal, such as “GEORGE” and “JEORGE” using edit distance metrics in Lucene.

Apache Lucene has its own query language that takes a search string as input with the following format: “<attribute name>: <text to be searched>”. Class QueryParser is used by CIDACS-RL to build the query and then IndexSearcher class retrieves the records that match the query. For example, an exact query with name and date of birth would have the following format:

$$\begin{aligned} +\hbox {name}:``<\hbox {name}>'' \quad +\hbox {dobirth}:``<\hbox {birthdate}>'' \end{aligned}$$

In Lucene’s query language, the character “$\sim$” is used to define fuzziness. In such type of query, Damerau-Levenshtein [32] is used as a distance metric and “$\sim$” means each query can return a similarity index of 0.5 or more. For example, an exact query with name, mother’s name and date of birth would have the following format:

$$\begin{aligned} name:``<\hbox {name1}\sim \quad \hbox {name2}\sim>'' \quad \hbox {dobirth}:``< \hbox {birthdate}\sim >'' \end{aligned}$$

By default, each query’s result in Lucene is ordered based on the normalized TF-IDF similarity index. The TF-IDF weight is composed by the normalized TF (the number of times a word appears in a document divided by the total number of words in that document) and the IDF (computed as the logarithm of the total number of documents divided by the number of documents where the specific term appears). Even though this metric is used to match strings on search domains, other edit distance metrics, such as Jaccard, Levensthein, and Jaro-Winkler perform better when matching names [34]. Hence, the TF-IDF in Lucene is used only for query and other methods are used for comparison.

CIDACS’ datasets also have date and categorical attributes that can be used for linkage. Some attributes may have semantic meaning which the TF-IDF does not account for; therefore, CIDACS-RL relies on a custom scoring function tailored for Brazilian data sources to compare record pairs. This function is based on different metrics and approaches, depending on the type of the attribute. CIDACS-RL supports four kinds of attributes: string, categorical, date, and municipality code/IBGE (“Instituto Brasileiro de Geografia e Estatistica/Brazilian Institute of Geography and Statistics”). IBGE code is a 7-digit numeric code where the first two digits represent one of Brazil’s 27 states, the following four digits represent one of 5570 municipalities, and the last digit is used for verification purposes.

**Fig. 2:**
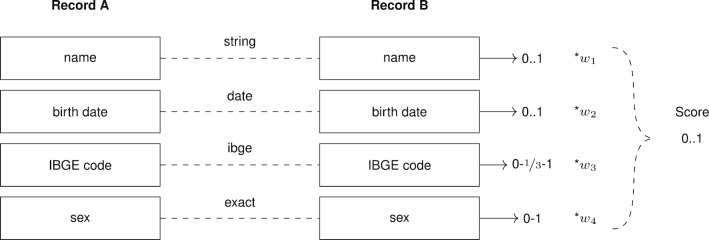
Example of a comparison between two records with four attributes. Each attribute is compared based on its type, generating a score between zero and one. Then, a set of weights defined empirically by the researcher is used to average the scores from the four attributes into a single final score

In CIDACS-RL, blocking is implemented by the indexing search function during the comparison process. All attributes present in the linkage are used for searching. Thus, records returned from the search function are very similar to records used as parameter to the search function. To compare a pair of records, CIDACS-RL first compares each pair of attributes present in those records. A set of empirically derived weights is passed as parameters to the system based on the discriminatory power of the attributes, which is used to summarize all scores into one value (Fig. 2). In order to derive the weights, we linked two databases such that the smallest can be manually reviewed multiple times (e.g., 200 records) and the initial weights were set with an educated guess. After running the linkage, we performed the manual review, calculated the frequency of true and false matches, and plotted the frequency of true and false matches for every threshold score to look for a threshold that divides the data well enough. This procedure was iterated by tweaking the weights until satisfactory results are obtained. The goal of tweaking the weights was to maximize the number of true matches above a threshold and minimize the number of false matches below this threshold.

Jaro-Winkler [34] was used to compare string attributes. Hamming distance, a metric comparing two binary strings of equal length as the number of bit positions in which the two bits are different, is used for date attributes. For categorical attributes, the comparison function set 1 for agreement and 0 for disagreement. In TF-IDF, the category in categorical variables such as gender and each piece of the text separated by space in string and date variables (for example, names and dates) were used as a term. For example, the name “Joao da Silva” becomes three terms: (Joao, da, Silva) and the date “01-01-2018” also becomes three terms: (01, 01, 2018).

#### Pairwise comparison

We used combined scoring and query modules to link every record in dataset *B* to a record in dataset *A*. Algorithm 1 shows the cascade approach used to combine the three kinds of search described in “Indexing and query processes” section *PairWiseComparison* function receives both the *Ai* index generated by the indexing module and the dataset *B*. Each query function (exact, semi-exact or fuzzy) takes each record in dataset *B* to query in *Ai* and returns a set of similar records based on TF-IDF (*similarRecordsArray*). Another function (*findMostSimilar*) uses the *score* function to compare the record in dataset *B* (which was used as a source for the query) with all records retrieved from *Ai* and finds the most similar record based on the custom scoring function. If any record with a score greater than the *threshold* is found by the exact query, the pair is added to the result and the semi-exact query is not executed. The same occurs for semi-exact and fuzzy. The steps described are performed for each record on dataset *B* and the function returns all pairs matched along with the score obtained. CIDACS-RL assumes that duplicates are removed earlier during data cleaning and standardization and, if still exist, the frequency is minimal given the nature of the datasets. In addition, when there is more than one record matched in the query, the algorithm selects the one that has the highest similarity score. Inconsistencies might arise if multiple records in B match best with one in A. This issue is addressed in the post-processing step by retaining the matches with the highest score. The code and implementation of CIDACS-RL is available on Github^1^ with example datasets. 
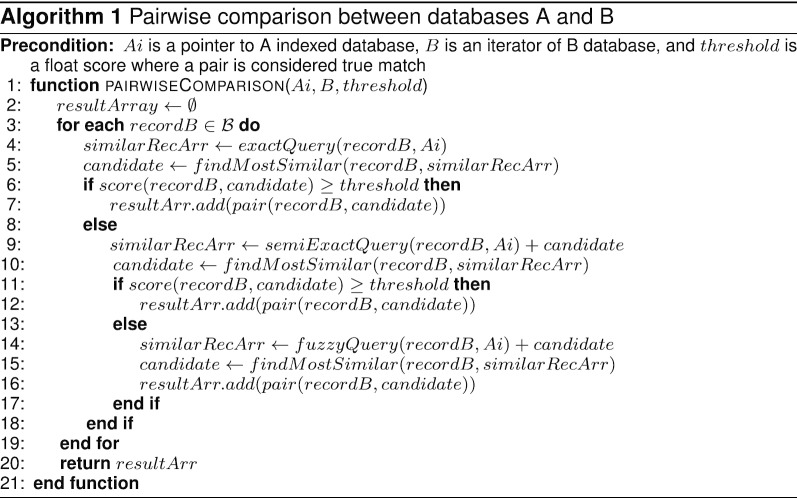


### Comparison with other linkage tools

We compared CIDACS-RL with other four well-established open source record linkage tools available in the literature: RecLink (version 3.1) [22], FRIL (Fine-Grained Records Integration and Linkage Tool - version 2.1.5) [35], Febrl (Freely Extensible Biomedical Record Linkage - version 0.4.2) [25], and AtyImo [36]. A gold standard dataset was used for comparison purpose. We used the same attributes available for all linkage methods and different configurations. In addition, as part of the experiment, the best case scenarios were pre-tested for each tool in order to find the best results for a fair comparison. Febrl, like CIDACS-RL, also provides blocking-indexing implementations, but during our comparison, the best results were achieved when running the system without blocking. Blocking was also required in RecLink, where the best blocking configuration was a combination of (first name of mother + municipality) and (last name of mother + municipality). In addition, different cut-offs are chosen for different linkage tools using ROC curve, since the linkage tools work differently, such that the maximum precision and recall are obtained. AtyImo, bespoke data linkage tool able to run over Spark and CUDA and implementing privacy-preserving techniques (through Bloom filters), was previously developed and initially used at CIDACS as part of a joint Brazil-UK project proposing the setup of the 100 Million Brazilian Cohort. However, its computational complexity (computational time) was prohibitive. CIDACS-RL brings advantages in terms of computational time although it assumes the existence of a safer room (no privacy-preserving techniques are required). A short description of these linkage tools compared in this section and selection of cut-off using ROC curve are provided in the Supplementary Material 1.

### Datasets

In this paper, we have used three different datasets to evaluate the performance of CIDACS-RL (summarized in Supplementary Material 2). We have derived a gold standard dataset from live births and mortality records to assess the performance of the different linkage tools. We also used a case study dataset, much larger than the gold standard dataset, from socioeconomic and demographics data linked to notifications of tuberculosis cases. This study was used to describe the performance of CIDACS-RL in big, real datasets. Finally, we created a synthetic dataset containing common attributes present in most Brazilian public health databases to assess the scalability of the CIDACS-RL tool. The case study and synthetic datasets were used only for CIDACS-RL. Detailed description of the data sets used to build the gold standard and case study datasets are summarised in the Supplementary Material 2.

#### Gold standard dataset

A gold standard dataset was created using two administrative data sources from the Brazilian Ministry of Health (MoH): the Mortality Information System (SIM, Sistema de Informações sobre Mortalidade) and the Live Birth Information System (SINASC, Sistema de Informações sobre Nascidos Vivos). Both SINASC and SIM contain individual-level data with five attributes in common: name, mother name, date of birth, municipality and sex. These attributes have been of good quality, in terms of completeness of recording, in the last decade. SINASC collect information on live births throughout Brazil using the “Statement of Live Birth” (DN — Declaração de Nascido Vivo) form with a unique identifying number, the DN number. SIM collects information on mortality using a death certificate. For children less than one year old, death certificate also includes the DN number.

**Fig. 3:**
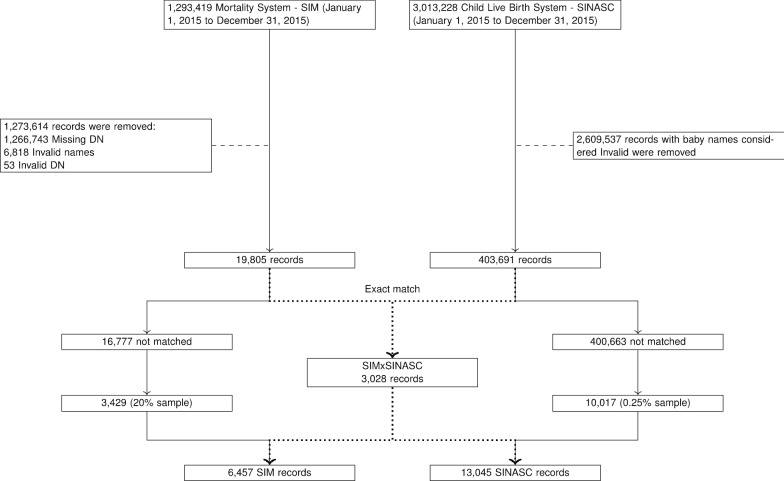
Construction of the gold standard dataset

From both datasets, records from one calendar year (2015) with complete and valid information on DN number and attributes were used. Then, the two datasets were linked using exact matching on the unique DN number, which is also used during the experiment to assess the performance of each tool. A number of non-linked records from SIM and SINASC were added to the final linked dataset in order to simulate a number of matches and non-matches usually found in CIDACS datasets (Fig. 3). Using this data, and after selecting cut-off points for each record linkage tool, sensitivity, specificity and PPV metrics were calculated for each of the five linkage tools: AtyImo, CIDACS_RL, Febrl, FRIL, and RecLink. Detailed description of gold standard is available in Supplemetary Material 2.

#### Case study: CadUnico x SINAN-TB dataset

This case study, used to assess the performance of CIDACS-RL tool in big real data, comprises data from two governmental databases: the Unified Registry for Social Programmes (CadUnico) and the Information System for Notifiable Diseases (SINAN, Sistema de Informações de Agravos de Notificação). This dataset was used only for CIDACS-RL to show its performance using huge dataset much larger than the gold standard dataset. Hence, the other linkage tools used on the gold standard dataset are not included here, in part because the tools do not run on such a size of dataset except AtyImo, at least in our experiment.

CadUnico is a database from the Ministry of Social Development built with the aim of identifying low-income families who could be eligible for social protection programmes, including conditional cash transfers (“Bolsa Família”), housing, and water wells (“Cisternas”). It has a wide range of demographic and socioeconomic information of more than 100 million records of Brazilian citizens, covering the period 2001–2015. The CadUnico dataset used in our experiment consolidated the whole period into a single dataset, which has 114,008,179 records (individuals). SINAN contains clinical cases of notifiable diseases collected by health professionals who attend patients with suspected diseases of compulsory notification within the Brazilian Public Health System (SUS). In this case study, we used a tuberculosis (TB) notification dataset from SINAN containing 1,182,777 reported tuberculosis cases from 2001 to 2013 in Brazil. Both datasets, CadUnico and SINAN, lack unique identifiers between them unlike the data sources used for the construction of the Gold Standard dataset for the comparison of the different linkage tools.

Five attributes (name, mother’s name, date of birth, gender, and municipality of residence) from each dataset were used to link these two datasets (CadUnico baseline with the SINAN-TB dataset). The dataset generated from the linkage procedure (all SINAN-TB records and their corresponding pairs in the CadUnico baseline) was analyzed to assess its quality, according to the following procedure:

A For each SINAN-TB record, there was a record in the CadUnico baseline which corresponds to the candidate pair with the highest score among all possible candidates. In addition, each record pair should contain information on the attributes used for linking the two datasets laid side by side;
B Manual verification was performed only for a random sample comprising 30,000 (94 excluded due to one/more invalid to missing attributes) hence, 29,816 pairs contained in the resulting dataset, since the total number of record pairs in the dataset was very large (n=1,182,777). The outcome of manual verification was established as a “gold standard” for further evaluating the performance of CIDACS-RL in linking these two datasets. Note that this “gold standard” is different from the gold standard dataset, described in the previous section, used for comparison of the five linkage tools.
C Sensitivity, specificity, positive predictive value and receiver operating characteristic (ROC curve) were estimated to assess the accuracy of the linkage.

#### Simulated datasets

Simulated datasets of 1-20 million records were obtained from [27] and are available on Github.^2^ There are five attributes in the datasets: name, mother’s name, sex, date of birth, and municipality of residence (IBGE code). Names were generated randomly based on a list of the most common names in Brazil. We tried to uniformly distribute dates of birth (from 1900 and 2010) and sex (‘M’ for male and ‘F’ for female) to generate a representative sample of the Brazilian population. Finally, municipality codes were picked from the full list of codes obtained from IBGE. The simulated datasets were used only to test the accuracy and scalability of CIDACS-RL when linking datasets of different sizes and potential diversity in their contents.

#### Performance metrics and statistical analyses

We assessed the accuracy of our linkage algorithms through standard metrics: sensitivity, specificity, positive predictive value (PPV), and area under receiver operating characteristic curve (AUC-ROC), as described in Supplementary Material 3. The main purpose of accuracy assessment in record linkage is identify how many matches and non-matches were retrieved by the linkage tools. True matches (also called true positives–TP) and true non-matches (also called true negatives - TN) are usually unknown prior to linkage. In our gold standard dataset, it was possible to know in advance all true matches and non-matches since we used the unique key identifier, the SINASC registration (DN) number to flag true matches. In the gold standard dataset, we also recorded the time taken (in minutes) to complete the linkage process.

We also assessed two types of errors that could appear during record linkage: false negative (FN), which is a missed match that can impact linkage sensitivity (i.e., a pair of records that should have been linked but were not), and false positive (FP), which is a false match and will impact PPV (i.e., a pair of records that should not have been linked because they are not the same but were incorrectly linked).

In the case study, we evaluated the performance of CIDACS-RL in huge databases larger than the gold standard dataset. We calculated the sensitivity, specificity, True matches, false matches, and missed matches for different thresholds.

Scalability of a record linkage tool is a critical challenge when dealing with huge datasets. The naive approach in record linkage involves a large number of comparisons equivalent to the product of the sizes of the two datasets; however, comparison of all record pairs using expensive functions has proven infeasible in most real-world applications [27]. To reduce this quadratic complexity, different linkage tools have implemented several blocking or filtering techniques. To assess the scalability of CIDACS-RL, we matched a single record to a larger dataset (simulated) and measured the time spent in this task. We tested the larger dataset starting from 1M records increasing by 1M for each execution until it reaches 20M records. For each dataset size, we executed the linkage tool 10 times and calculated the arithmetic mean of the execution times.

We compared two scenarios: (i) the linkage was performed serially, using only one processor (single core), and (ii) we adapted CIDACS-RL to run over Spark [37] and used Spark’s pseudo-distributed mode to parallelise the execution and run over eight logical cores. The hardware used for this experiment was a Intel i7 4770 with 16GB of RAM.

## Results

### Gold standard dataset

**Fig. 4:**
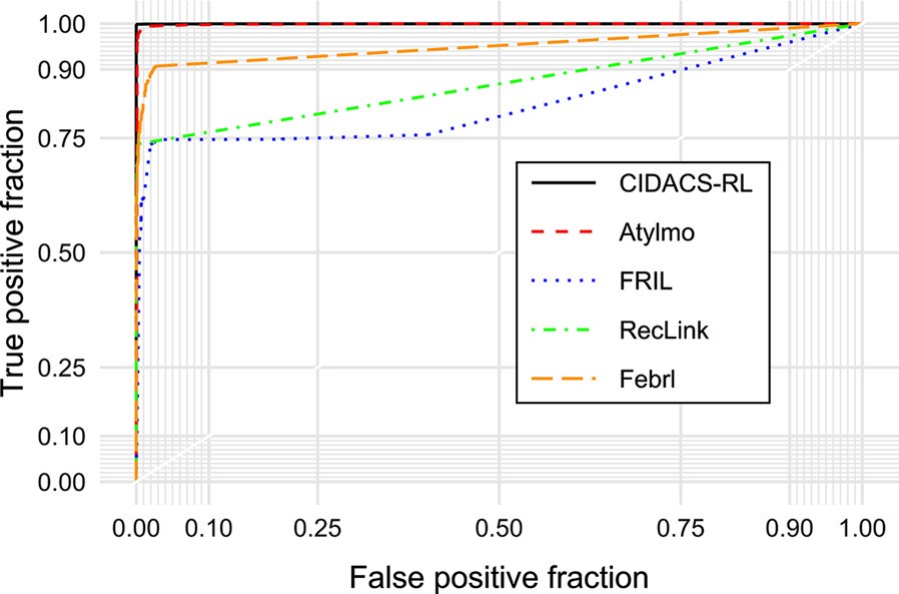
ROC curves for each record linkage tool over the gold standard dataset

Since each linkage tool produces a dataset containing a similarity score for each pair, ROC curves were plotted to find the best cut-off point that maximizes accuracy for each tool. Figure 4 presents the ROC curves for each record linkage tool compared with the gold standard dataset. CIDACS-RL had higher AUC-ROC compared to the other linkage tools.

Table 1 presents the comparative performance amongst the different record linkage tools using the gold standard dataset. Cut-off points were selected for each tool and the sensitivity, specificity and PPV metrics were calculated based on these values. CIDACS-RL has higher sensitivity (99.94%) and PPV (99.93%) compared to the other linkage tools.

**Table 1:** Threshold analysis for each record linkage tool

Method*	Threshold (TH)	Pairs above TH	Sensitivity	Specificity	FPs above TH	FNs below TH(%)	PPV
CIDACS-RL	0.8827056	3026 (46.86)	99.87	99.94	2 (0.07)	4 (0.13)	99.93
AtyImo	8777	3005 (46.54)	98.91	99.39	21 (0.70)	33 (1.09)	99.30
RecLink	0.8075590	2243 (34.74)	73.75	99.71	10 (0.45)	795 (26.25)	99.55
Febrl	3722604	2832 (43.86)	90.58	97.40	89 (3.14)	285 (9.41)	96.86
FRILL	48	2351 (36.41)	74.66	97.36	90 (3.83)	767 (25.33)	96.17

*Execution time (in minutes): CIDACS-RL < 1, AtyImo = 28, RecLink < 1, FRIL = 7, and Febrl = 130

### Case study: CadUnico x SINAN-TB dataset

In the case study dataset. after manual verification of the sample, 17,355 pairs of records were identified as true matches and 12,461 as false matches. Based on this result, we analyzed accuracy of CIDACS-RL in huge dataset through ROC curves to identify an appropriate cut-off point to classify matched pairs, as summarized in Table 2. The optimal cut-off point (0.896) was chosen after varying this value between 0.86 and 0.93 and assessing specificity and sensitivity results. Using this cut-off point, the 95% confidence interval estimated for sensitivity (92.5%), specificity (93.5%), and area under the ROC curve (97.2%) for CIDACS-RL were 92.07–92.99, 93.08–93.8, and 96.97–97.35, respectively.

Applying this cut-off point (0.896), pairs of records in the complete dataset were classified as linked if their scores were greater than or equal to the cut-off point and not linked if their scores were lower.

**Table 2:** Case study (CadUnico x SINAN-TB) dataset: linkage analysis

Cut-off	Specificity	Sensitivity	Matches (%)	True matches (%)	False matches (%)	Missed true matches (%)
0.860	75.0	97.1	16,443 (55.15)	12,100 (73.59)	4343 (26.41)	361 (2.90)
0.870	82.2	95.5	14,984 (50.25)	11,901 (79.42)	3083 (20.58)	560 (4.49)
0.880	87.7	94.5	13,901 (46.62)	11,770 (84.67)	2131 (15.33)	691 (5.55)
0.890	91.8	93.3	13,046 (43.76)	11,621 (89.08)	1425 (10.92)	840 (6.74)
0.896	93.5	92.5	12,661 (42.46)	11,532 (91.08)	1129 (8.92)	929 (7.46)
0.900	94.2	91.7	12,423 (41.67)	11,424 (91.96)	999 (8.04)	1037 (8.32)
0.910	95.8	89.8	11,931 (40.02)	11,194 (93.82)	737 (6.18)	1267 (10.17)
0.920	96.7	88.1	11,546 (38.72)	10,972 (95.03)	574 (4.97)	1489 (11.95)
0.930	98.0	85.4	10,984 (36.84)	10,636 (96.83)	348 (3.17)	1825 (14.65)

Description of tuberculosis (TB) notifications as true matches, false matches, and missed matches using the different thresholds is presented in Table 2. Overall, we found 42.50% of tuberculosis cases correctly linked to the CadUnico dataset and 8.9% of the tuberculosis cases were incorrectly linked to the CadUnico dataset. We also checked for biases in the linked data on four dimensions of race/skin color, sex, age groups, and new cases. All but sex showed similar distributions in the linked and non-linked data. Sex had a bias in the linked data with more females in the linked data (27.84%) compared to the non-linked data (42.53% ). Detailed descriptive analysis of the demographic characteristics of linked and unlinked records in the case study: CadUnico x SINAN-TB linkage is presented in Table 1 of Supplementary Material 4.

### Simulated dataset

**Fig. 5:**
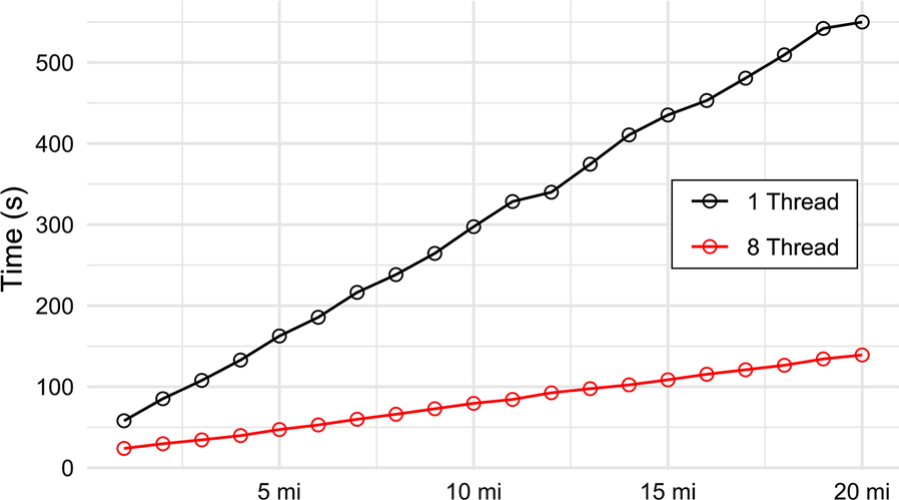
Scalability tests using two different hardware setups on pseudo-distributed mode of Spark

Figure 5 shows the execution times for CIDACS-RL using one thread (serial) and eight threads (parallelised) systems. Large differences in performance were observed as the size of the datasets increases. The parallelised execution was about four times faster than the serial when using the 20M database (150 seconds versus 550 seconds). We have tried to compare the performance of other linkage tools used in the gold standard dataset; however, the other linkage tools except AtyImo were not able to run with datasets of such sizes. Hence, the scalability result was reported only for CIDACS-RL.

## Discussion

CIDACS-RL has proved to be a very powerful and accurate tool both in controlled (using the gold standard dataset) and uncontrolled (using the case study datasets) experiments. The novelty presented by this tool is the combined implementation of indexing search and scoring as a blocking step for record linkage. Such implementation aims to improve speed, scalability, and accuracy over huge datasets where common unique key attributes are not available, such as the 100 Million Brazilian Cohort. Compared to AtyImo, a linkage tool previously developed at CIDACS, CIDACS-RL has shown superior accuracy, measured using positive predictive value and sensitivity, and a shorter execution time.

The current implementation of CIDACS-RL is an iterative deterministic linkage based on five attributes and involving different queries (exact, semi-exact and fuzzy) in the pairwise comparison step. The semi-exact query, like the classical deterministic linkage algorithms, was developed to work with a small number of columns ($<10$), which seems sufficient for most real-world linkage applications. The use of more linkage attributes ($\ge 10$) might increase the number of searches leading to prolonged execution time and more complexity when choosing thresholds; however, its potential impact needs to be evaluated quantitatively and weighted against any gains from inclusion of more attributes. Previous studies have reported that step-wise deterministic linkage has better performance compared to simple deterministic algorithm which requires exact matching on all attributes [38–40]. The benefit of achieving higher PPV outweighs the cost of relatively lower sensitivity as the size of the dataset (particularly the larger one) increases [41]. This is similar to our settings in CIDACS where the baseline (CadUnico) dataset, to which other datasets are linked, is substantially huge (>114 million records).

Finding a convenient and accurate method for linkage performance validation in the absence of a gold standard dataset and choosing an appropriate cut-off when using manual review require a careful decision since it heavily impacts the classification of matches and non-matches.The use of machine learning techniques have been suggested for automated optimization of linkage parameters which also ensures human errors are reduced. For example, artificial neural networks and clustering algorithms can also be used to deal with missing data and produce accurate results with maximized F-measure [42–45].

Privacy-preservation (anonymization) is another critical challenge in record linkage initiatives involving big health datasets which contain massive amounts of personal and sensitive data. In its current version, CIDACS-RL does not implement privacy preservation techniques; however, we aim to use a similar approach implemented in AtyImo to provide such capability in the future. While privacy preservation is required through the entire linkage process, it has proved difficult to find a linkage tool that optimizes the main linkage challenges (quality, privacy, and scalability). For example, high linkage quality and/or privacy could be achieved through computationally complex approaches such as secure multi-party computation techniques, machine learning techniques or graph-based approaches; however, these methods might not be scalable to large databases [46–48]. Privacy enhanced manual review process, to improve quality without too much compromise on privacy, designed to work in conjunction with automatic RL methods has been suggested [49–51]. Bloom filters [26], binary vectors of size *n* initialized with zeros, using hash functions are very reliable alternatives with high scalability and accuracy, and they were implemented in AtyImo [27]. Development and evaluation of accurate, scalable and privacy-preserving linkage techniques will remain an open area for future research.

Our linkage tool has several strengths. It is very fast hence has reasonably short execution time compared to other linkage tools; it can link large databases with tens of millions of records over standard computers without the need for high speed processors; and it is scalable to distributed infrastructures. In addition, it has high accuracy and sensitivity compared to other open source linkage tools. This is mainly due to the implementation of Lucene’s indexing search and scoring instead of classical blocking and filtering methods. The later usually do not use all attributes present in linkage datasets for indexing [52], which can cause totally different records to be compared and hence wastage of computational resources. Further improvements in processing time and accuracy can be achieved through the implementation of machine learning tools and using customized search function instead of Lucene’s default TF-IDF approach to score the search.

The optimal choice between deterministic and probabilistic linkage methods needs consideration of several factors that influence performance, which include database quality, availability of unique identifiers, file size, acceptable trade-offs between positive predictive value and sensitivity for a specific linkage project, and resource availability (software programs and high speed computers). More importantly, data standardization, cleaning, flexibility on approximate matches are important in both approaches. For example, when there is substantial missing values and/or error in the linkage attributes, both methods may not be suited for linkage. A future work is planned to extend the CIDACS-RL with probabilistic implementation and compare it to the current step-wise deterministic version.

## Conclusion

CIDACS-RL algorithm utilizes combined indexing search and scoring for linking huge datasets that pose tremendous computational challenges to other linkage tools. This innovative application of existing technologies instead of traditional blocking has resulted in higher accuracy, scalability, and substantially shorter execution time when compared to other linkage tools. The tool can also be employed on standard computers without the need for high speed processors and it is also scalable to parallelised or distributed infrastructures. It has made possible linking CIDACS’ huge datasets with tens of millions of records in few days to build large cohorts such as “the Brazilian 100 Million Cohort” within the CIDACS environment. This enables epidemiological research on associations and impact of social protection policies on a large range of health outcomes, with a degree of detail (individual level) never done before. Further developments of the tool are currently in progress.

## Supplementary information

**Additional file 1:** Description of: (1) the different linkage tools; (2) the datasets and the creation of the gold standard dataset; (3) the comparison metrics; (4) the descriptive analysis of the linkage between CadUnico and SINAN-TB; (5) the determination of Threshold in Weight Vector Classification.

## Data Availability

The code and implementation of CIDACS-RL is available on Github with example datasets. https://github.com/gcgbarbosa/cidacs-rl-v1

## Abbreviations

AUROC: Area under receiver operating characteristic curve
CadUnico: Cadastro Único
CI: Confidence interval
CIDACS: Centro de Integração de Dados e Conhecimentos para Saúde (“Centre for Data and Knowledge Integration for Health”)
CIDACS-RL: CIDACS record linkage
DN: Declaração de Nascido Vivo (“Statement of Live Birth”)
DSA: Dataset A
DSB: Dataset B
DSCnds: Dataset candidate pairs
DSIndex: Indexed dataset A
DSResult: Dataset result
Febrl: Freely extensible biomedical record linkage
FN: False negative
FP: False positive
FRIL: Fine-grained records integration and linkage tool
GPUs: Graphics processing units
IBGE: Instituto Brasileiro de Geografia e Estatística (“Brazilian Institute of Geography and Statistics”)
MoH: Ministry of health
PPV: Positive predictive value
RAM: Random-access memory
ROC: Receiver operating characteristic
SIM: Sistema de informações sobre mortalidade (“Mortality Information System”)
SINAN: Sistema de Informações de Agravos de Notificação) (“Information System for Notifiable Diseases”)
SINASC: Sistema de Informações sobre Nascidos Vivos (“Live Birth Information System”)
TB: Tuberculosis
TF-IDF: Term frequency-inverse document frequency

## Footnotes

1 https://github.com/gcgbarbosa/cidacs-rl-v1.

2 https://github.com/spiros/atyimo.
